# Three-Dimensional-Printed Photopolymer Resin Materials: A Narrative Review on Their Production Techniques and Applications in Dentistry

**DOI:** 10.3390/polym17030316

**Published:** 2025-01-24

**Authors:** Özge Mine Yüceer, Esra Kaynak Öztürk, Elif Su Çiçek, Nagehan Aktaş, Merve Bankoğlu Güngör

**Affiliations:** 1 Department of Prosthodontics, Faculty of Dentistry, Gazi University, Ankara 06490, Türkiye; mineyuceer@gmail.com (Ö.M.Y.); kynkesra03@gmail.com (E.K.Ö.); elifsucicek97@gmail.com (E.S.Ç.); 2 Department of Pediatric Dentistry, Faculty of Dentistry, Gazi University, Ankara 06490, Türkiye; nagehanaktas@gazi.edu.tr

**Keywords:** 3D printing, additive manufacturing technologies, dental materials, digital dentistry, dental resin

## Abstract

Additive manufacturing (3D printing) has transformed dentistry by providing solutions with high precision and accuracy achieved through digital workflows, which facilitate the creation of intricate and personalized structures. Additionally, 3D printing promotes cost efficiency by reducing material waste and errors while enabling on-demand production, minimizing the need for extensive inventories. Recent advancements in 3D-printed resin materials have enhanced their clinical applications by improving mechanical strength, biocompatibility, esthetics, and durability. These innovations have facilitated the fabrication of complex and patient-specific structures, such as dental prostheses, surgical guides, and orthodontic appliances, while significantly reducing production time and material waste. Ongoing research and innovation are expected to strengthen resin properties, including strength, translucency, and durability, broadening their clinical applications. The ongoing evolution of 3D printing technology is poised to play a critical role in driving personalized treatments, streamlining clinical workflows, and shaping the future of dental care. This narrative review comprehensively examines the production techniques and clinical applications of 3D-printed photopolymer resins across various dental specialties, including prosthodontics, orthodontics, pediatric dentistry, maxillofacial surgery, periodontology, endodontics, and conservative dentistry. Additionally, the review provides insight into the transformative impact of these technologies on patient care, highlights existing challenges, and suggests future directions for advancing resin properties and their integration into routine dental practice.

## 1. Introduction

Additive manufacturing (3D printing) technologies have revolutionized dentistry by enabling precise, cost-effective, and customizable solutions for various applications [1]. These technologies offer numerous advantages in dentistry, making them a transformative tool for modern dental practice and laboratory workflows. One of the most significant benefits is the precision and accuracy achieved through digital workflows, allowing for creating highly detailed and customized dental structures. These technologies streamline production by eliminating the need for traditional molds and manual adjustments, significantly reducing material waste and production time. The ability to fabricate complex geometries and intricate designs enhances the functionality and fit of dental devices, improving patient comfort and clinical outcomes. 3D printing also supports cost-effectiveness by minimizing errors and material usage while enabling on-demand production, reducing the need for extensive inventories. The use of biocompatible materials, combined with the versatility of different 3D printing techniques, has further expanded the scope of dental applications [2,3,4]. Adopting this technology involves significant costs due to the investment required in various software and hardware [5]. The widespread success of 3D printing is mainly due to its cost efficiency and the reduced time required compared to traditional methods [6]. Studies have shown the capability to deliver more predictable and cost-effective treatments with 3D printing technologies [7,8]. Daher et al. [7] presented a clinical report on the 3D printing workflow to rehabilitate worn dentition. They reported that definitive composite resin restorations produced through 3D printing are considered a more cost-effective and less technique-sensitive alternative [7]. In another study, Daher et al. [8] aimed to assess the integrity of the marginal adhesive interface of an initial formulation of a 3D-printed composite resin before and after thermal and mechanical fatigue, as well as to evaluate the efficiency of this manufacturing method. The 3D-printed resins offered lower equipment and consumable costs and reduced production time, particularly when fabricating more than eight restorations [8].

The accuracy of the digital workflow and conventional methods has been investigated in several studies [9,10,11,12]. Luu et al. [9] compared the accuracy of digital and conventional cross-mounting by assessing 3D deviations at each step of sequential cross-mounting. They reported that digital cross-mounting demonstrated greater accuracy than conventional methods, though deviations were higher in the anterior region compared to the posterior. Palantza et al. [10] reported that the impression method (conventional or digital) and the choice of impression material in conventional techniques significantly influence the accuracy of impressions for inclined and parallel implants. It was suggested that digital impressions were reliable but should be used cautiously for full-arch implant restorations. A systematic review of 79 studies examined the accuracy of implant impressions. Among these, 59 studies compared various conventional impression techniques, 11 focused on different digital impression methods, and 9 evaluated the two approaches. The findings indicated that digital impressions provided accuracy comparable to conventional methods. Notably, conventional techniques were less accurate for impressions of edentulous arches and implants with inclinations, whereas digital methods were unaffected by these challenges [11]. Another review analyzed 20 studies, including 5 in vivo and 15 in vitro. Several studies used conventional impression techniques as controls, with open-tray impressions and polyether being the most common. The results consistently demonstrated that digital impressions were as accurate or, in some cases, more precise than conventional methods [12].

**Figure 1:**
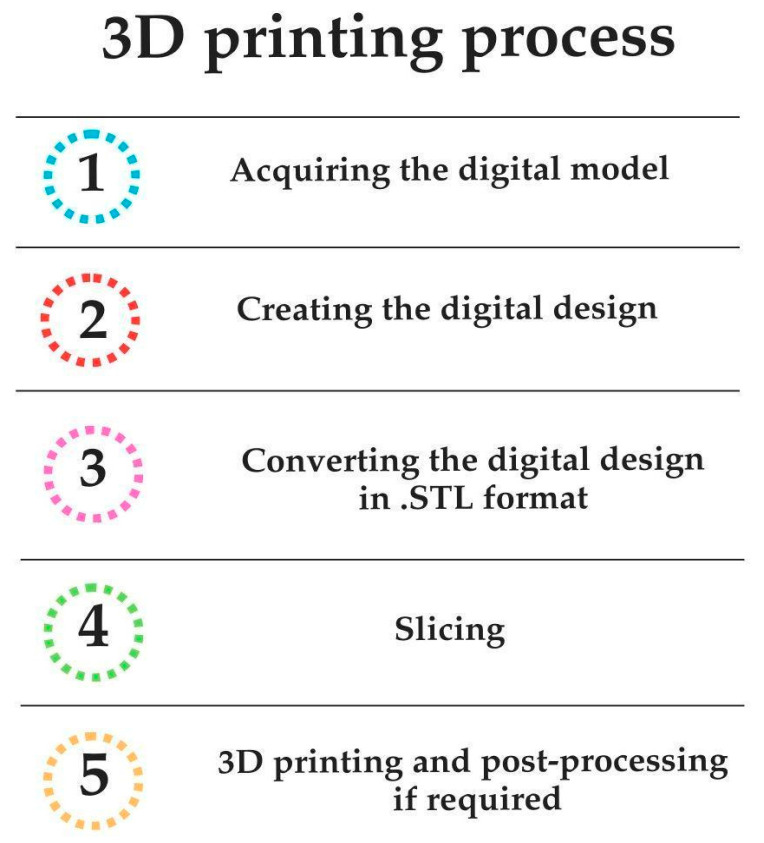
The sequential steps involved in the 3D printing process for dental applications begin with data acquisition through digital scanning techniques and progress to virtual design, fabrication using additive manufacturing methods, and final post-processing.

Applying additive manufacturing technologies in dentistry involves well-defined steps integrating digital workflows with precise fabrication techniques. The process begins with acquiring a digital model, typically generated through intraoral scanning, tomography images, or extra-oral scanners. These data are then used to create a virtual design of the desired dental restoration or appliance using computer-aided design (CAD) software. Then, the design is converted into a format compatible with the 3D printer, such as standard tessellation language (STL) files. The selected additive manufacturing method then uses materials to build the object. Post-processing steps ensure the final product meets clinical and esthetic requirements, including cleaning, curing, and finishing. This streamlined process improves accuracy and customization and reduces production time, enabling dentists and labs to deliver high-quality results efficiently [3,4,13,14]. The steps of the 3D printing process are summarized in Figure 1.

Three-dimensional models obtained through various methods are divided into countless 2D layers for additive manufacturing using software programs, and the data are transferred to the additive manufacturing device for production. The additive manufacturing device converts the raw material into a thin solid layer along the x-y axis. Subsequently, it creates a new layer by moving within a specified interval along the z-axis, maintaining the construction cycle until the production is complete [15,16]. The additive manufacturing method produces restorations layer by layer by selectively focusing on specific portions of the material using applications such as lasers [17,18]. This approach reduces the excessive material consumption commonly encountered in subtractive manufacturing. Enabling the reuse of unused waste materials saves approximately 95–98% [19]. However, additional support materials are required [17]. Material waste from 3D printing, including failed prints, byproducts, and supports, poses environmental challenges due to the limited recyclability of the polymers used. Most plastics from stereolithography and material jetting processes are non-recyclable. To address this, systems for returning waste materials to suppliers for recycling should be established, and designs should minimize the need for supports to reduce waste generation [5]. Producing directly from CAD models eliminates the need for other physical models, further reducing costs. The digital nature of the design files also facilitates modifications and enhances customizability [19]. The properties of the subtractive and additive manufacturing are summarized in Table 1.

The rapid advancement of 3D printing technology has revolutionized dentistry, offering unprecedented opportunities for customization and precision. Among these innovations, 3D-printed dental resins have garnered significant attention for their ability to facilitate the fabrication of tailored dental prostheses, orthodontic devices, and restorative materials. Numerous types of 3D-printed resins have been developed, each tailored to meet the demands of specific dental applications [20]. While previous studies have predominantly focused on the technical processes or the applications within particular fields, such as orthodontics or pediatric dentistry, this review adopts a comprehensive approach [4,7,14,15,21,22]. This review aims to provide a holistic overview of the manufacturing techniques and clinical applications of 3D-printed resins across all subdisciplines of dentistry, emphasizing their transformative impact on patient care and clinical workflows.

## 2. Production Techniques of the 3D-Printed Photopolymer Resins

The American Society for Testing and Materials (ASTM) has established seven categories for additive manufacturing: vat photopolymerization, binder jetting, material jetting, powder bed fusion, directed energy deposition, material extrusion, and sheet lamination [23].

**Figure 2:**
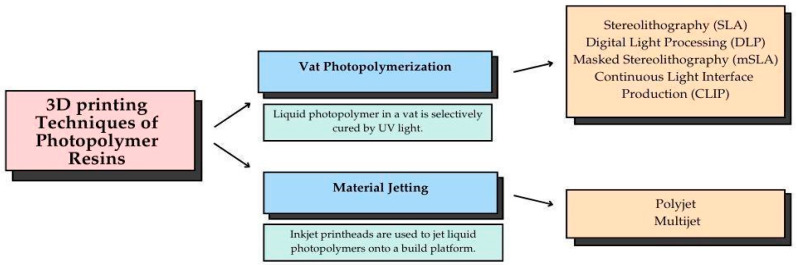
The key 3D printing techniques of photopolymer resins, including vat photopolymerization and material jetting, and a comparative overview of their working principles [26,27,28,29,30].

Polymers are considered the most widely used materials in 3D production due to their material diversity and easy adaptability to different methods. Polymers are available for 3D production in thermoplastic filaments, reactive monomers, resin, or powder [24,25]. The most commonly used form of 3D-printed resins in dentistry is photopolymers, and vat polymerization and material jetting 3D printing manufacturing techniques are generally used in the production processes. The manufacturing techniques of the 3D-printed photopolymer resins are summarized in Figure 2 [23].

### 2.1. Vat Photopolymerization

Vat photopolymerization is the process of creating a structure by polymerizing liquid photopolymer resin layer by layer with the help of ultraviolet light from a vat [31]. Several factors, such as the type of light source, the material properties, and the overall printing procedure, influence the carbon footprint and energy usage of vat polymerization. Generally, vat polymerization is energy-intensive due to the high energy required for light sources, such as lasers or LCD screens, to cure the resin. However, ongoing advancements in material efficiency and light-source technology are helping to reduce the energy consumption of this method. Vat polymerization shows considerable potential for mass production, especially in sectors like automotive, healthcare, and dental restorations, where high precision and complex designs are needed. The main challenge for scalability involves lowering material costs, increasing printing speed, and improving post-processing efficiency. Moreover, developing sustainable and biocompatible resins aims to mitigate the environmental impact associated with vat polymerization [32,33,34,35].

The photopolymerization technique, known for producing intricate parts and structures with superior accuracy and resolution, has gained popularity due to its advantages, including precision and rapid processing [36]. The main disadvantage of vat photopolymerization technologies is the restricted range of materials available. Multi-material printing presents significant potential by combining diverse chemical components into a single material with unique properties distinct from those of individual components. However, achieving multi-material vat photopolymerization requires a comprehensive understanding of the photochemistry underlying the polymerization process [37].

**Figure 3:**
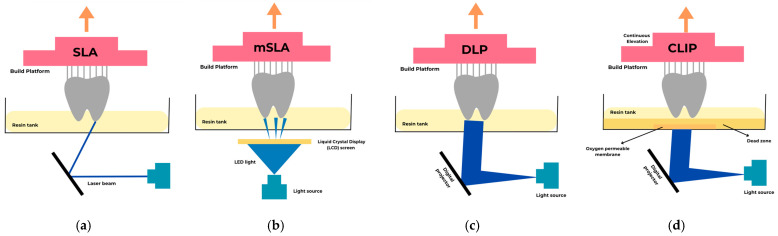
The working principles of vat-polymerization printers commonly used in dentistry include (**a**) SLA, (**b**) mSLA, (**c**) DLP, and (**d**) CLIP. It highlights the mechanisms of each technique, demonstrating their approaches to layer-by-layer fabrication, light-source configurations, and resin curing processes.

However, multi-material vat photopolymerization 3D printing is still in its early stages. It faces several challenges, including (1) limited monomer functionality, (2) a restricted range of photopolymers compatible with multi-material printing, (3) loss of reaction orthogonality, (4) material leaching and difficulties in maintaining long-term properties, and (5) issues with the viscosity and printability of precursors at suitable resolutions [37]. The most common vat-polymerization techniques are SLA, mSLA, DLP, and CLIP. The working principles of the vat-polymerization printers used in dentistry are summarized in Figure 3.

Chuck Hull’s invention of the stereolithography (SLA) method is considered the first stage in the development of 3D printing technologies [31,38]. SLA is commonly employed in various industries to produce complex models, high-quality jewelry, detailed prototypes, dental and medical devices, and custom components [25]. SLA is known for producing highly accurate designs and is frequently used in dentistry for creating temporary and permanent crowns, fixed partial dentures, surgical guides, templates, and diagnostic casts and models [14]. The photopolymerization system initially used offers advantages such as enhanced resolution, smooth surfaces that often require no post-processing, strong chemical bonding between layers resulting in good *z*-axis strength, and fast production [14,29]. The working mechanism of SLA begins with constructing a platform to stabilize the part and support overhanging structures. Each layer is exposed along the *x*-*y* axis, with adjustments possible, while the *z*-axis incrementally advances throughout the fabrication process [39]. A build platform is submerged into liquid resin for SLA production and then polymerized using a UV laser. A scanning mirror guides a focused laser beam into a reservoir of UV-sensitive resin, solidifying each layer with precision [14,29].

The laser outlines the cross-section of each layer. Once a layer is cured, the build platform descends by a distance equal to the layer’s thickness, enabling a fresh coat of uncured resin to cover the previous layer. This process is repeated multiple times until the printed object is entirely constructed. Between layers, the platform lowers by 50 µm or less for high-resolution applications or 200 µm or more for parts requiring lower resolution. After the part is completed, any excess resin can be drained and reused. The printed parts are cleaned to eliminate residual resin, and the support structures are manually removed [18,39,40]. Additionally, some devices use a bottom-up photopolymerization approach. These devices feature a UV-transparent, non-stick platform and gradually raise the inverted build platform during the process, minimizing the need for support structures [39].

In the SLA process, the curing depth, which defines the *z*-axis resolution, is regulated by the photoinitiator and the irradiation exposure parameters, including wavelength, power, and exposure duration. This allows for resolutions of 10 µm in both the x-y plane and the *z*-axis [18,39,40]. Precision in the dimensions of the printed object is essential to ensure proper fit and functionality, especially for crowns, fixed partial dentures, and aligners [41]. With the rapid advancement of technology since the initial introduction of SLA, various improvements have been made in recent years. In the first-generation laser-scanning SLA systems, galvanometric mirrors directed the laser along the *x*-*y* axis, often resulting in defocusing and optical errors. To mitigate these issues, a fixed laser system with an x-y translation stage and a transparent glass window was developed to control resin layer thickness. While this method enabled the movement of either the optical system or the printing platform, it introduced challenges with resin adhesion to the glass. To overcome this, researchers developed a free-surface system that prints objects directly onto the platform, eliminating adhesion problems [36,42]. The quality of printed structures in SLA depends on the cure kinetics of the polymerization process, which is influenced by factors such as light intensity, illumination time, resin viscosity, chemical functionality, and the additives in the formulation. Photoinitiators initiate the polymerization reactions, while light absorbers enhance the resolution of the printed objects. SLA technology is constrained by the limited availability of photopolymers. Additionally, the cytotoxicity and irritation potential of the resins and unreacted monomers in printed objects must be carefully considered when using SLA 3D printing [37]. The productivity advantage of SLA can offset its slower unit printing speed, which is not the case with DLP or LCD printers [29].

In recent years, a new production method known as masked stereolithography (mSLA) has been introduced among SLA printers. Masked stereolithography (mSLA) is a 3D printing technology that cures liquid synthetic resins using UV light. Unlike standard stereolithography, which uses lasers, mSLA employs LEDs as the light source. The LED light is directed through a liquid crystal display (LCD), and each layer is cured using a masking technique. The LCD screen selectively blocks unwanted areas, ensuring the accurate formation of cross-sections. The resolution of mSLA depends on the pixel size of the LCD screen; however, practical resolution loss may occur due to light diffusion. During the printing process, the build platform moves upward after each layer is cured, and the part is progressively formed. After printing, the part is washed with isopropyl alcohol to remove excess resin and cured under UV light for complete solidification. One drawback of mSLA technology is that the high light intensity of the LCD screens can cause overheating, leading to rapid screen degradation. Although these printers are generally more cost-effective than SLA and DLP printers, they have ergonomic limitations and lack optimized print profiles specifically designed for dental applications. The primary advantage of mSLA lies in its ability to produce complex geometries with high precision and speed. This technology is particularly suitable for applications in dentistry, such as the fabrication of prosthetic restorations, delivering good surface quality. However, post-processing can be time-consuming, material costs remain high, and multi-material printing capabilities must be available [29,43]. Depending on the brand, the cost of LCD printers is 2- to 10 times lower than SLA or DLP printers despite having comparable prices. This difference is partly due to lower manufacturing costs and the limited optimization of non-dental-specific LCD printers. These printers are generally less ergonomic and lack certified printing profiles [29]. Precision in LCD printing is partly compensated for by an algorithm, but the technology suffers from optical convergence issues. For smaller printing volumes, DLP theoretically achieves higher trueness than SLA due to its high-resolution projection on small surfaces, surpassing the SLA laser’s resolution. However, SLA is generally considered more precise than DLP and LCD, although this precision primarily impacts surface finish rather than adaptation or insertion quality. LCD printing appears less accurate than DLP, but the accuracy of LCD 3D printing has not been extensively studied. Low-cost LCD printers are often less ergonomic than high-cost models, relying on open-source software and lacking automated printing and post-processing features. Furthermore, specific printing parameters for each resin are not always provided and may need to be determined by the user [29].

ASTM classifies digital light processing (DLP) as being in the same additive manufacturing category as SLA because the technologies share many similarities [18,23]. This technique is used in rapid prototyping, jewelry casting, dental applications, and creating customized consumer goods [25]. In SLA, the light source is a laser, whereas in DLP, the light source is an arc lamp or a semiconductor chip that includes a matrix of microscopic mirrors. This allows SLA systems to cure a layer through a pen-like drawing process, while DLP cures the entire layer at once, creating restorations layer by layer through polymerization [18,40]. The chip used in DLP is also called a micro-mirror device because it contains hundreds of thousands of tiny mirrors that can move in two directions and switch on and off thousands of times per second. This feature allows DLP printers to create a model in voxels instead of layers, significantly enhancing the surface finish quality [40]. The equipment’s mapping system improves production efficiency, measurement accuracy, surface quality, and the ability to tailor printed objects by modifying photocurable resin formulations. However, its projectors mainly emit broad-spectrum light, with a significant portion falling below 400 nm, which is less effective for activating photoinitiators [30]. A major disadvantage of DLP technology lies in the size of each voxel. Larger voxel sizes result in lower resolutions, producing blockier and more angular designs, while smaller voxels lead to higher resolutions and smoother results. DLP printing, however, currently produces clinically acceptable temporary and permanent restorations, including crowns, fixed partial dentures, and removable prosthetic devices [14]. Additionally, DLP is less prone to oxygen inhibition compared to SLA. This is because the resin layer is polymerized consistently at the bottom of the vat, reducing its direct exposure to ambient air [41].

Continuous liquid interface production (CLIP) is another 3D printing technique where photosensitive material is illuminated with smooth model transitions as the table steadily lifts the model from the resin vat. High resin reactivity and light intensity are crucial to this process [44]. CLIP utilizes LEDs and oxygen to enhance printing speed and resolution [45,46]. Similar to DLP technology, it incorporates a dead zone between an oxygen-permeable window and the curing surface. This zone prevents adhesion to the window and inhibits free radical photopolymerization, ensuring smooth operation. A crucial element in continuous 3D printing is the vat bottom material, which must be light-permeable and prevent resin polymerization at the contact surface [46,47]. This method has limitations, particularly with large, flat horizontal surfaces. The combination of viscous resin and continuous movement generates significant suction forces between the bottom membrane and the printout during lifting, leading to membrane deformation and model distortion [48]. The minimum dead-zone thickness for CLIP-based 3D printing has been determined to be around 20 to 30 μm. Since the dead zone is influenced by print parameters, working below this threshold can increase the likelihood of defects caused by window adhesion. It was indicated that printing microstructures required highly accurate settings, often involving dynamic adjustments tailored to 3D CAD. This has created a growing need for advanced numerical simulations capable of predicting the CLIP printing process and providing guidance for optimizing print parameters. Such simulations can help reduce trial-and-error, decrease material waste, and enhance print quality [49].

### 2.2. Material Jetting

**Figure 4:**
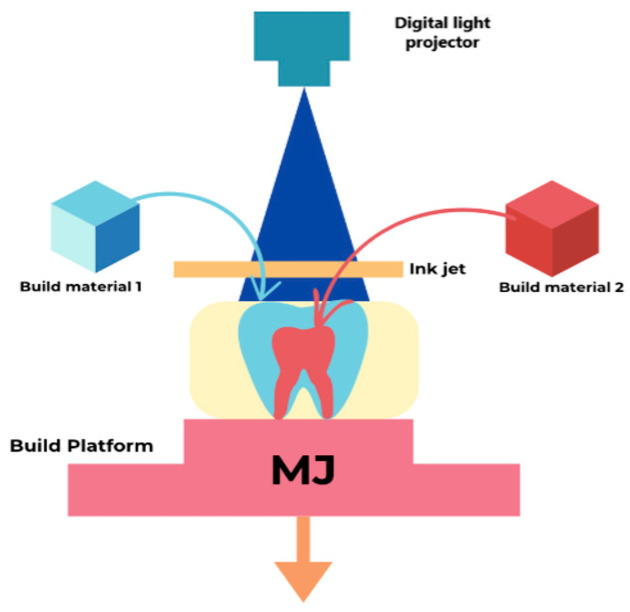
The working principle of material jetting printers used in dentistry highlights the layer-by-layer deposition and curing process.

Material jetting (MJ), or photopolymer jetting, works by depositing tiny droplets of material onto the build platform through a print head, followed by intermediate photopolymerization. This method eliminates the need for post-polymerization [50]. MJ is a technology based on material deposition and photopolymerization. It can be defined as the process of depositing liquid photopolymer droplets onto a designated area, which are then cured using UV light to form a layer. This process is repeated until the entire product is fabricated. The typical layer thickness in MJ is approximately 16 to 32 µm [51]. Parts produced with this method offer several advantages over those manufactured through other processes, including homogeneous mechanical properties, dimensional accuracy, excellent surface quality, and low surface roughness [52,53]. Additionally, MJ production allows for using various materials, such as acrylics, photopolymers, metals, and ceramics, with diverse colors and physical properties [30,54]. The working principles of the material jetting printers used in dentistry are summarized in Figure 4.

The first step in MJ involves heating the liquid resin to 30 °C and 60 °C to achieve the optimal viscosity. The next step entails the movement of the print head, which deposits tiny photopolymer droplets onto the required locations on the build platform. The UV-light-equipped print head cures the deposited photopolymer, forming the object’s first layer. Once a layer is printed, the build platform moves downward by the height of a single layer, allowing the next layer to be printed. These steps are repeated until the final object is complete [51]. Although MJ equipment and materials are costly, removing support materials can be challenging [22]. MJ includes two similar technologies: PolyJet and MultiJet. PolyJet and MultiJet technologies are similar but differ significantly in post-processing. PolyJet uses a photocurable material for support, while MultiJet employs wax. This distinction impacts the removal process, making the post-processing procedures of Polyjet shorter and more straightforward than MultiJet [55,56]. PolyJet (also known as Inkjet) is well-suited for rapidly producing multi-material models using multi-nozzle jetting, which also allows for support materials. However, using shear-thinning materials limits the range of available materials [24]. PolyJet technology selectively jets UV-curable resins onto a build platform, curing each layer as it is deposited. This method enables the fabrication of multi-material prints with a range of colors and Shore A hardness values in a single process. PolyJet 3D printers offer enhanced creative possibilities using multi-color materials [57]. PolyJet printing produces multi-material and multi-color components, making it well-suited for dental models and temporary crowns. However, its limited mechanical properties reduce its suitability for oral applications, and frequent print-head clogs pose maintenance challenges. Nonetheless, its capability to create highly esthetic, multi-color prints positions it as a promising technology for advancing dental applications [14]. A major disadvantage of PolyJet printing is the need for regular maintenance of the print heads, which are prone to clogging. Currently, PolyJet is used to fabricate dental models. However, the material lacks strong mechanical properties, limiting its advantages in the oral environment. Despite this, photopolymer jetting printing shows great potential for transforming the dental industry, particularly in esthetic dentistry [14].

The advantages and disadvantages of vat polymerization and material jetting are listed in Table 2.

## 3. Applications of 3D-Printed Photopolymer Resins in Dentistry

In dentistry, 3D-printed resins have revolutionized various specialties by improving precision, efficiency, and clinical outcomes through patient-specific solutions. In prosthodontics, 3D-printed resins fabricate temporary and permanent crowns, inlays, onlays, veneers, and fixed and removable prostheses with exceptional accuracy and esthetics [2,59]. In orthodontics, they enable the production of clear aligners, retainers, and customized appliances such as maxillary expanders and brackets, offering enhanced fit, reduced treatment time, and improved patient comfort. In maxillofacial surgery, resins create highly accurate surgical guides, splints, and anatomical models, facilitating precise implant placement and reconstructive procedures [17,60]. For pediatric dentistry, 3D-printed resins produce biocompatible crowns and durable space maintainers, which provide esthetic advantages and streamline clinical workflows [21]. In conservative dentistry, 3D-printed injection guides simplify composite restorations, improving precision and reducing chair time [61], while in endodontics, patient-specific guides aid in navigating complex root-canal anatomies and apical surgery, enhancing treatment predictability [15,62]. In periodontology, 3D-printed resins are pivotal in regenerative therapies through bioresorbable scaffolds, membranes, and esthetic gingival surgeries with customized surgical guides [63,64]. Moreover, their role in research and education is significant, with dental models and replicas aiding in the simulation of clinical scenarios and procedural training [65].

The 3D-printed structures produced with vat polymerization require post-processing treatment. Post-processing involves successive isopropyl alcohol baths to eliminate unpolymerized resin, followed by post-polymerization in a UV oven to achieve the resin’s optimal properties. These steps are outlined by the manufacturer to ensure the desired quality in terms of mechanical properties and biocompatibility [66].

The 3D-printed photopolymerized resins should have sufficient mechanical properties for using them in dental applications. The flexural strength of most cured 3D-printed resins met ISO standards, even after aging [67]. The minimum required flexural strength values were 65 MPa for denture base resins [67], 50 MPa for interim resins [68,69], and 50 MPa for orthodontic appliances [70]. The strength of 3D-printed resin materials is influenced by pre-printing, printing, and post-printing stages. Pre-printing factors involve enhancing the resin by adding reinforcing agents. Printing factors include parameters such as printing orientation, layer thickness, third-party material use, and objects’ positioning on the printing platform. Post-printing factors encompass post-curing conditions (time, temperature, and curing device), post-rinsing duration, finishing and polishing techniques, and storage practices [65]. Additives are used to increase the strength of 3D-printed denture base resin, including zirconia nanoparticles, titanium dioxide nanoparticles, zinc oxide nanoparticles, silver nanoparticle-loaded cellulose nanocrystals at low concentrations, silicon dioxide nanoparticles, essential oil microcapsules, and nanocarbon [71,72,73,74]. Furthermore, zirconia nanoparticles were incorporated into 3D-printed interim fixed prostheses, while animated nanodiamonds were added to 3D-printed resin for orthodontic appliances [71,75,76]. Besides mechanical properties, the surface and optical properties of the 3D-printed resins were chosen to be improved. Morel et al. [77] investigated the addition of 5% aramid fibers on the mechanical, surface, and optical properties of the 3D-printed denture base materials. The color difference (∆E_00_) was below the perceptibility threshold (≤1.1). It was stated that adding aramid fibers into 3D-printed resin for denture bases enhanced mechanical properties without significantly affecting surface characteristics. AlGhamdi et al. [78] evaluated the effects of zirconium dioxide and silicon dioxide nanoparticles on the hardness, surface roughness, and color stability of 3D-printed provisional restorations. Two resins (ASIGA and NextDent) were modified with 0.5 wt% and 1 wt% of the nanoparticles, while one group remained unmodified. Zirconium dioxide and silicon dioxide nanoparticles significantly increased hardness compared to unmodified resins, with 1% zirconium dioxide nanoparticles yielding the highest value but no significant difference between the two nanoparticle types. Surface roughness was not affected, with 1% zirconium dioxide nanoparticles in NextDent showing the highest roughness value. Zirconium dioxide nanoparticles caused higher color changes (ΔE_00_: 4.1–5.8), while silicon dioxide nanoparticles induced minimal changes (ΔE_00_: 1.01–1.85). It was concluded that incorporating the tested nanoparticles improved hardness without affecting surface roughness, but zirconium dioxide nanoparticles significantly impacted optical properties.

Resins intended for contact with the oral tissues must be biocompatible and comply with current standards. In Europe, these standards are defined by the European Union (EU) Regulation 2017/745 on the classification of Medical Devices (MD) and the International Organization for Standardization (ISO) 10993 standard for biocompatibility [79]. A key issue with polymers is the release of cytotoxic monomers due to incomplete polymerization. These residual monomers can cause allergic reactions, irritation, and sensitization of the oral mucosa, and may even exhibit genotoxic and reprotoxic effects. These effects were linked to the degree of monomer-to-polymer conversion, emphasizing the need to improve this conversion rate to enhance resin biocompatibility [66]. In a systematic review, Cabrol et al. [66] evaluated the effect of post-processing on 3D-printed resin biocompatibility in prosthodontics in 27 studies. In those articles, 32 commercially available and some experimental resins were tested, primarily for applications such as denture bases, denture teeth, interim and definitive fixed restorations, occlusal splints, and surgical guides. It was concluded that most post-processed 3D-printed resins were found to be non-cytotoxic, indicating adequate biocompatibility, and the dentist is responsible for following the manufacturer recommendations.

**Figure 5:**
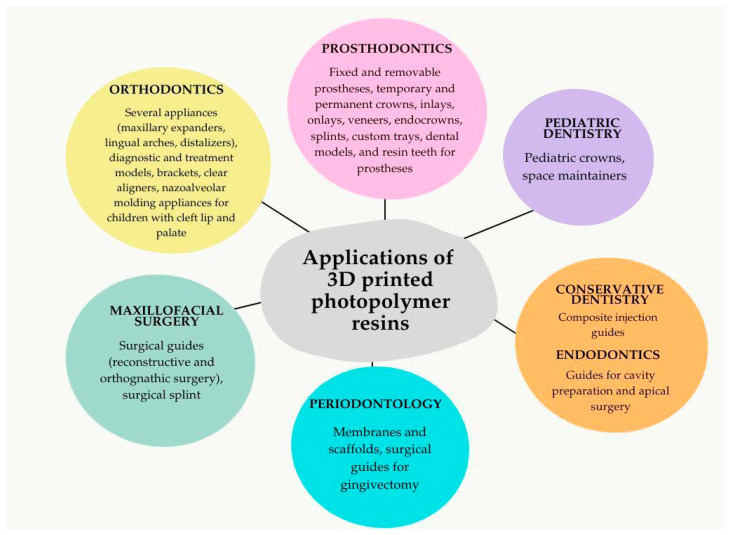
Areas of specialties (prosthodontics, orthodontics, maxillofacial surgery, pediatric dentistry, periodontology, endodontics, and conservative dentistry) and applications of 3D-printed photopolymer resins in dentistry.

With continued advancements in resin properties, including improved strength, wear resistance, and translucency, the scope of 3D-printed resins in dentistry continues to expand [14]. The applications of 3D-printed resins in dentistry, categorized by dental specialties, are summarized in Figure 5.

Commercially available 3D-printed photopolymer resins, production technologies, application areas, and manufacturers are listed in Table 3.

### 3.1. Applications of 3D-Printed Resins in Prosthodontics

The introduction of 3D printing technologies has revolutionized the production of fixed and removable prostheses, making the process more patient-friendly and efficient. These technologies offer numerous advantages in prosthetic dentistry, including improved time efficiency, enhanced precision for compatible restorations, repeatability, cost-effectiveness, user-friendliness, and superior mechanical properties. The flexibility to produce complex geometries and patient-specific designs contributes to their growing adoption in prosthetic dentistry [2].

Three-dimensional-printing technologies, such as SLA and DLP, are widely used for producing temporary and permanent crowns, inlays, onlays, veneers, endocrowns, and fixed partial prostheses. Resin-based 3D printing materials, including photopolymers like methacrylate-based dental resins, are commonly employed due to their ability to achieve high-resolution and smooth surfaces. These resins are designed to provide biocompatibility, sufficient strength, and esthetic properties [148]. The scientific data on 3D-printable materials for permanent restorations in restorative and prosthetic dentistry remain sparse. Key challenges include the absence of standardized testing protocols, material shortcomings such as insufficient strength and wear resistance, and limited long-term clinical studies. Future priorities should involve creating standardized testing methods, improving material performance, undertaking comprehensive clinical trials, and defining clear regulatory frameworks. With these advancements, 3D printing is poised to become an integral part of routine dental practice [149].

For removable prostheses, including complete and partial dentures, 3D printing technologies enable the production of custom-fit prostheses with excellent adaptation to the oral cavity. SLA and DLP are commonly used, employing resins specifically formulated for denture bases and teeth. These materials meet ISO standards for material strength and biocompatibility. Studies have shown that 3D-printed removable prostheses exhibit comparable base adaptation and durability to conventionally fabricated prostheses [59,150]. Enhancing mechanical strength, aging resilience, and esthetic appeal, along with improving retention and advancing printing technology for higher resolutions and precise occlusal adjustments, are key to optimizing 3D-printed denture base materials [59,74].

Traditional prosthetic reconstruction techniques often face challenges in accurately recreating complex defects in facial prostheses. However, it has been reported that these limitations can be overcome using modern 3D printing technology. This technology primarily fabricates prosthetic frameworks and negative molds for facial prostheses. The primary materials used include resins and resin waxes [3,151].

Moreover, 3D printing technology is particularly well-suited for producing custom impression trays. CAD software enables the quick and precise design of trays with optimal fit parameters, including the virtual blocking of undercuts to prevent irreversible deformation during tray removal. However, the high cost of materials for functional impression trays limits their use primarily to implant impression trays. Combining this approach with digital implant planning is recommended, as digital models can guide tray fabrication based on the planned implant positions [152].

Notably, 3D printers, known for their high efficiency and precision, are widely used for digitally fabricating master and segmented models. These systems are particularly valuable in oral implantology, where the precise positioning of laboratory analogs in the printed model is critical. Accurate placement ensures the optimal proximal and occlusal fit of restorations, making this an essential application for DLP printing technology [152].

Biocompatible dental resins can be used for removable dentures, mimicking traditional acrylic resin properties, and permanent denture teeth are made from methacrylate-based photopolymerized resin, processed via 3D printing. The denture base and teeth are printed separately and bonded with a light-cured agent, followed by an additional polymerization step for durability [153]. Furthermore, 3D printing transforms complete denture fabrication, offering the potential for cost-effective, in-office production that could reshape the market. While clinical workflows benefit, laboratory workflows still demand skilled design, printing, and finishing, limiting accessibility for many clinicians. Innovation is focused on enhancing resin materials to improve physical and esthetic properties. Although ideal printed denture materials are yet to be developed, the current options meet ISO standards for strength and base adaptation. Challenges such as fractures, color stability, and staining persist but are expected to improve as material technology advances, marking the start of an exciting future for printed dentures [81].

### 3.2. Applications of 3D-Printed Resins in Orthodontics

In orthodontics, 3D printing provides numerous benefits, including enhanced customization, reduced production time, and improved precision. It enables orthodontists to develop patient-specific appliances that optimize treatment efficiency and outcomes while minimizing waste and resource use [60]. In recent years, 3D printing technologies have enabled designing and producing highly customized orthodontic applications [154]. These appliances include maxillary expanders, lingual arches, and distalizers. In addition to diagnostic and treatment models, brackets and clear aligners are produced in orthodontics with 3D printing [15,60,155].

Using a fully digital workflow, 3D printing technologies allow for creating patient-specific bracket systems. These brackets are designed to offer improved fit, reduce treatment duration, and enhance overall treatment outcomes [60,156,157,158]. In the study by Waldman et al., the clinical efficiency and efficacy of 3D-printed brackets were compared to conventional bracket systems. The 3D-printed group experienced a 45% reduction in average treatment time compared to the conventional group. Additionally, patients with 3D-printed brackets required approximately six fewer scheduled appointments and exhibited fewer loose brackets. The study concluded that a 3D-printed bracket system enhances clinical efficiency by significantly reducing treatment duration and the number of appointments needed while maintaining effective treatment outcomes [159].

Additionally, 3D-printed resins for indirect bonding trays have emerged as a transformative approach in orthodontics, offering high precision in bracket placement. By leveraging CAD–CAM technology, these trays facilitate accurate bracket positioning on dental models, reducing treatment duration and chair time while improving patient comfort. Studies have highlighted that shell-design trays demonstrate more significant clinical acceptability limits than bar-design counterparts, with deviations well within acceptable thresholds [160,161]. The study by Mahran et al. [116] compared the accuracy of 3D-printed indirect bonding trays to vacuum-formed trays for orthodontic bracket placement. The study concluded that 3D-printed trays are generally more accurate than vacuum-formed trays in linear dimensions, with similar angular control and immediate debonding rates (10.7% vs. 7.1%, respectively). The authors concluded that 3D-printed trays provide higher precision and are preferable for indirect bonding procedures. In the study conducted by Schwätzler et al. [162], the transfer accuracy and immediate loss rate of hard and soft 3D-printed indirect bonding trays were evaluated. The mean transfer errors were reported as −0.162 mm and 0.255° in the hard-resin group and −0.011 mm and 0.243° in the soft-resin group. The immediate loss rates were 2.4% for the hard-resin group and 2.3% for the soft-resin group. The researchers concluded that soft trays offer better usability and accuracy.

Clear aligners, commonly used to treat mild malocclusion or prevent relapse after fixed orthodontic treatment, have seen rapid advancements with 3D printing. This technology is crucial in producing aligners by creating accurate dental models for thermoforming aligner sheets. Aligners produced through additive manufacturing demonstrate superior wear resistance and anatomical accuracy compared to those made with traditional thermoforming techniques. Furthermore, advancements in 3D-printer technologies and resin materials have supported the growing application of clear aligners in clinical practice, offering patients a more comfortable and efficient treatment experience [117,157,163].

Of note, 3D printing technologies have also proven valuable in treating children with cleft lip and palate. Digital models of the patient’s oral cavity allow for virtual treatment planning, guiding surgical and orthodontic interventions. For nasoalveolar molding, 3D-printed appliances can help align the alveolar structure before surgical lip repair, improving both function and esthetics. These appliances provide precise and patient-specific solutions, reducing the need for manual adjustments during treatment [132,164]. In the study conducted by El-Ghafour et al. [164], the effectiveness of a newly introduced 3D-printed nasoalveolar molding appliance in improving maxillary arch dimensions in infants with unilateral complete cleft lip and palate before surgical lip repair was investigated. Maxillary-arch dimension measurements performed on digital models by blinded assessors revealed significant clinical and statistical improvements in the 3D-printed group compared to the control group. The study concluded that using the 3D-printed nasoalveolar molding appliance is a simple, efficient method to enhance maxillary arch dimensions before surgical lip repair.

Integrating 3D printing into cleft lip and palate repair represented a major advancement in personalized medicine, enabling tailored treatments and improving surgical precision, speech and hearing outcomes, operative times, and education. Future developments in automation, artificial intelligence, and augmented reality promise further innovation. However, challenges such as cost-effectiveness, ethics, and accessibility must be addressed to enable broader adoption in reconstructive surgery [165]. Virani et al. [166] evaluated the 3D printing in cleft care in a systematic review. In the review, a total of 39 articles were included. The primary focus was on the applications of 3D printing in cleft lip and palate care, emphasizing its advantages over traditional methods. Secondary outcomes included cost analysis and clinical outcomes. The included articles were categorized into six primary applications of 3D printing: nasoalveolar molding, patient-specific implants, bioprinting, surgical planning, surgical simulation/training, and educational or anatomic models [166]. Nasoalveolar molding emerged as the most common application of 3D printing in cleft lip and palate care; 3D printing streamlines the nasoalveolar molding process by reducing time, effort, and cost for both providers and patients [132,167,168]. By utilizing scans of infant maxillary impressions, molds can be digitally modified and produced through additive manufacturing, creating multiple nasoalveolar molding devices from a single mold [167,169]. There is a notable lack of comprehensive cost analyses for 3D printing in caring for patients with cleft lip and palate. While certain applications, such as nasoalveolar molding, surgical training and simulation, and educational models, have reported costs that support broader adoption, challenges remain. These include the absence of standardized protocols, process variability, and significant upfront cost barriers, which may limit the reproducibility of 3D printing applications. However, in an era focused on patient satisfaction, 3D printing offers a competitive edge for providers, potentially justifying the associated expenses [166].

### 3.3. Applications of 3D-Printed Resins in Maxillofacial Surgery

Advancements in 3D imaging technologies, such as computed tomography (CT), have improved diagnostic precision and treatment planning in oral and maxillofacial surgery. Combined with 3D printing technologies, this progress offers advantages such as time and cost efficiency, the enhanced mechanical strength of surgical implants, greater accuracy and precision, improved esthetics, and reduced surgical time and risk. Integrating 3D printing technologies in maxillofacial surgery enhances precision, shortens operation time, and improves patient-related outcomes [2,170,171,172].

Moreover, 3D printing is crucial in producing patient-specific preoperative and intraoperative surgical guides based on CT data. These guides are designed to fit specific bone segments, facilitating precise cutting or drilling during surgery. By being tailored to the patient’s anatomy, they provide significant advantages for both the patient and the surgeon [4,173]. Typical applications include reconstructive surgery for maxillofacial tumors or trauma, orthognathic surgery to address skeletal malocclusion, and surgeries aimed at restoring occlusion and facial esthetics [3].

Surgical splints, often used in jaw surgeries to stabilize dental occlusion, are produced as replicas of the patient’s final postoperative anatomy. Materials used in 3D-printed splints, such as biocompatible transparent resins, are designed for excellent compatibility with the patient’s unique anatomy, offering improved fit and reduced postoperative complications. SLA technology is commonly employed in fabricating these splints due to its high precision and ability to work with biocompatible materials [173]. The implementation of 3D-printed maxillofacial surgical splints customized to patient anatomy has greatly improved surgical outcomes, particularly in precision, efficiency, and patient satisfaction. It was stated that these personalized splints enhance surgical accuracy by up to 30%, minimizing deviations from planned surgical paths and ensuring precise alignment during procedures. Additionally, operative durations have been reduced by an average of 15–25%, owing to the tailored preoperative design and effective intraoperative guidance offered by these splints. Patient satisfaction has also shown notable improvement, with over 90% of patients reporting comfort and satisfaction with the custom fit, which has contributed to faster recovery times and fewer postoperative complications [174,175,176].

### 3.4. Applications of 3D-Printed Resins in Pediatric Dentistry

Advancements in 3D printing technology have revolutionized pediatric dentistry by providing innovative and patient-friendly solutions. These technologies enable the production of customized pediatric crowns and space maintainers with reduced material wastage, faster production times, and fewer patient visits [21].

Also, 3D-printed resins are increasingly used to fabricate pediatric crowns, providing an esthetic and durable alternative to traditional stainless-steel and prefabricated zirconia crowns. SLA and DLP technologies are commonly employed to produce these crowns using biocompatible resin materials. These resins offer optimal strength, gingival compatibility, and esthetics, making them ideal for primary teeth restorations. Studies have demonstrated that 3D-printed resin crowns provide better marginal fit, improved gingival health, and higher patient satisfaction than traditional crowns [177,178]. Al-Halabi et al. [178] conducted a randomized clinical trial to compare 3D-printed resin crowns and CAD–CAM-fabricated PMMA crowns for restoring pulp-treated primary molars. They found that both crown types demonstrated acceptable outcomes in terms of marginal integrity and crown retention. However, 3D-printed crowns showed fewer cementing failures and better gingival health at the 12-month follow-up. In another study by Al-Halabi et al. [177], the clinical outcomes of 3D-printed resin crowns and direct composite crowns were compared as restorations for primary molars. At the 12-month follow-up, no significant differences were observed in crown failure rates between the groups. However, 3D-printed crowns demonstrated superior gingival health at 6 and 12 months and better marginal integrity across all evaluation periods, while direct composite crowns exhibited higher retention rates. Lee et al. [179] evaluated the clinical performance of 3D-printed resin crowns as an esthetic alternative to stainless-steel crowns for restoring primary molars after pulp treatment. Over a 12-month follow-up, no significant differences were found in the plaque index, but the gingival index and occlusal wear were significantly higher in the 3D-printed group. While survival rates were 100% for stainless-steel crowns and 82.1% for 3D-printed resin crowns, resin crowns offered aesthetic superiority and repairability. Parents’ esthetic satisfaction with 3D-printed resin crowns was very high at follow-up, and most of them opted for RCs when the other teeth needed to be fully restored [179].

It is well-known that 3D-printed resin crowns are designed to withstand the biting forces of children and offer faster production times [86,87,88,89,105]. Aktaş and Bankoğlu Güngör [86] conducted a study to assess the fracture resistance of permanent resin crowns for primary teeth produced using two different 3D printing technologies (DLP and SLA) and cemented with various luting cements. The study found that DLP-printed crowns exhibited higher fracture resistance compared to SLA-printed crowns. These crowns were determined to withstand the masticatory forces in children [86]. In a study by Aktaş and Bankoğlu Güngör [88], the enamel wear and fracture resistance of six types of esthetic pediatric crowns were evaluated: prefabricated zirconia, prefabricated composite, milled composite, milled resin matrix ceramic, milled PEEK, and 3D-printed resin crowns. The study concluded that all crown types tested are capable of withstanding masticatory forces in children, with material-specific differences in wear and fracture resistance [88].

Furthermore, 3D printing technologies have also been transformative in producing space maintainers, critical for preserving dental arch integrity after a premature loss of primary teeth. A benefit of 3D-printed space maintainers is that they overcome the limitations of traditional designs, such as solder joint failures, by integrating the band and loop into a single unit. This eliminates weak points, enhances durability, and provides excellent fit and contact pressure distribution, reducing long-term complications like bone resorption. These devices also offer shorter production times and fewer patient visits, making them a more efficient option for pediatric patients [85,180,181,182]. In an in vitro study by Aktaş and Atabek [85], the fracture resistance of band and loop space maintainers produced with 3D-printable materials (metal, resin, and PEEK) was evaluated and compared to conventional space maintainers after thermal aging. The results showed that 3D-printed metal, resin, and PEEK space maintainers demonstrated clinically acceptable fracture-resistance values. Among these, resin space maintainers were highlighted as a preferable option due to their superior esthetic properties [85].

### 3.5. Applications of 3D-Printed Resins in Conservative Dentistry

Moreover, 3D printing technology has introduced significant advancements in the restoration of anterior teeth by providing non-invasive and efficient solutions. Specifically, 3D-printed guides for anterior restorations enable the precise transfer of designs created in software to the patient’s oral cavity. These guides simplify direct composite restoration procedures, reduce chair time, and help achieve the expected esthetic outcomes, including proper color matching and anatomical accuracy [61,138,183,184].

Moreover, 3D-printed composite injection guides are gaining popularity as a precise and efficient alternative to conventional silicone mock-up methods for anterior restorations. These guides are fabricated using patient-specific designs created in CAD software and translated into physical models through high-resolution 3D printing [48,138,183,184]. In the study conducted by Zhang et al. [61], the effect of 3D-printed injection guides was evaluated using a fully digital workflow for the closure of post-orthodontic interdental spaces. With this technique, the gaps in the anterior teeth of a 15-year-old patient were closed and it was reported that satisfactory clinical results were provided in the short-term follow-up. The study reported that free-hand composite restorations offer an approach that provides faster and more predictable results, as this method is time-consuming and sensitive to technical precision [61]. The study presented by Watanabe et al. [183] introduced a flexible 3D-printed resin index equipped with a stabilization holder. This innovative system was developed to provide better stability and accuracy during the restoration of teeth. The flexible 3D-printed resin index used was obtained from a digitally designed dental wax-up, and the stabilization holder ensured the index fit perfectly to the tooth surface. The study reported that the method significantly simplified the clinical processes and reduced technical precision. It was also stated that this approach increased the predictability of restorative outcomes and offered esthetic results. The technique was successfully applied to restore the maxillary lateral incisor of an 18-year-old male patient with microdontia, achieving improved esthetics and function [183]. Puri et al. [184] presented a clinical report describing a fully digital workflow for restoring an impacted canine using 3D-printed reduction guides and a resin-injection index. The workflow included digitally designed and 3D-printed diagnostic wax-ups, tooth reduction guides, and resin-injection indices. This minimally invasive approach allowed for precise tooth preparation and the reshaping of the canine into a central incisor, improving esthetics and enabling effective space management during orthodontic treatment. The report highlighted the predictability and success of digital dentistry in achieving long-term provisional restorations with high precision and aesthetic outcomes [184]. Shui et al. [138] introduced a novel composite injection technique using a 3D-printed template with an interproximal isolation design to address multiple anterior diastemas in succession and evaluated the clinical results of the technique in a case with a 10-month follow-up. In contrast to traditional methods, this approach was reported to provide precise and effective restorations by integrating interproximal matrices to prevent resin extrusion. The mechanical properties of the rigid 3D-printed template were reported to significantly reduce chair time and technical precision by enabling accurate transfer from digital wax-up to intraoral restorations and to provide predictable and esthetically pleasing results [138].

### 3.6. Applications of 3D-Printed Resins in Endodontics

Overall, 3D printing technology has significantly enhanced the precision and predictability of endodontic procedures by providing patient-specific solutions. From guided cavity preparation to apical surgery, 3D-printed guides and tools offer innovative solutions for navigating complex root-canal anatomies and improving surgical accuracy [15,62,185].

One of the critical challenges in root-canal therapy is accessing the canal system, particularly in teeth with complex or calcified root canals, and 3D-printed guides have been shown to facilitate locating and accessing these canals with greater precision. Micro-guided endodontics is an emerging concept that combines minimally invasive drills (0.85 mm diameter) with 3D-printed surgical templates. This approach provides a predictable solution for treating calcified root canals, especially in anterior teeth, and has been reported to reduce operating time and improve clinical outcomes [3,185,186].

In apical surgery, 3D-printed guides enhance the accuracy of osteotomy and root-end resection. These guides, produced with data from CBCT scans, allow for the precise targeting of the surgical site, even in challenging anatomical cases such as palatal roots of maxillary first or second molars, roots of mandibular premolars adjacent to the mental nerve, and mandibular molars with thick buccal bone plates. Studies have shown that combining trephine drills with 3D-printed guides increases surgical accuracy compared to traditional endodontic microsurgery [2,187,188].

Moreover, 3D-printed training models developed using advanced resin technology have reportedly revolutionized endodontic education by providing realistic simulations of clinical scenarios. The use of durable, customizable resins has been reported to enable the precise replication of dental anatomy, allowing students to practice complex procedures such as working length determination and root-canal filling in conditions close to clinical conditions. It has been stated that this approach will reduce costs in the long term while increasing students’ preparation and confidence for real-world endodontic treatments. In endodontics and other branches of dentistry, 3D-printed training models are increasingly being adopted, providing realistic simulations for different procedures [65,135,136,137].

### 3.7. Applications of 3D-Printed Resins in Periodontology

In periodontology, 3D printing technology has become essential, focusing on regenerative periodontal therapies and esthetic gingival surgeries. By providing customized solutions, 3D-printed membranes, scaffolds, and surgical guides are advancing the precision and effectiveness of periodontal treatments [63,64,189,190].

In regenerative periodontics, 3D-printed membranes and scaffolds are critical in promoting tissue regeneration and enhancing bone resistance to occlusal forces. These biomaterials are designed to maintain the structural and functional integrity of the oral cavity. Produced using SLA/DLP technologies and materials such as polylactic acid (PLA) or polycaprolactone (PCL), bioresorbable scaffolds accurately replicate the anatomical organization of periodontal tissues. Bioprinting further enhances this process by enabling the creation of complex morphologies with precise biological functionality [63,189,190]. Periodontal regeneration remains challenging despite advancements, with therapies like guided tissue regeneration showing limited efficacy in fully restoring the periodontium. Promising approaches, such as periodontal ligament stem cell-based therapies and biomimetic scaffolds, face technical and regulatory hurdles, necessitating advanced models replicating the periodontal ligament’s mechanical environment using 3D printing and bioreactors. Collaborative, multidisciplinary efforts are crucial to developing reliable, standardized solutions for engineering functional periodontal ligament for clinical use [191]. Mohd et al. [192] evaluated in vivo and in vitro studies on 3D-printed scaffolds for dental applications. In vitro studies highlighted their ability to support cell viability, enhance cellular functionality, promote differentiation, and regulate the expression of tissue-specific markers, essential for successful tissue engineering. In vivo evaluations in animal models demonstrated the potential of these scaffolds to regenerate bone, periodontal tissue, dentin, and pulp, providing a foundation for advancing clinical applications in dental and craniofacial regeneration [192]. In another systematic review, Figueiredo et al. concluded that multi-compartment, fiber-guiding, and ion-containing 3D-printed scaffolds have demonstrated superior outcomes in true periodontal regeneration in animal models compared to traditional scaffolds or negative controls. These advanced scaffolds enhance tissue organization and regeneration by mimicking the natural periodontal structure and delivering ions that promote cellular activity, highlighting their potential for more effective periodontal therapy [193]. Davidopoulou et al. [194] assessed multidimensional 3D-printed scaffolds for intrabony periodontal defect regeneration, and they noted that multidimensional, 3D-printed customized scaffolds show promise in stimulating periodontal regeneration. However, further research is needed to determine the optimal scaffold characteristics for effective and reliable periodontal tissue regeneration. Three-dimensional bioprinting offers significant advantages, including design flexibility and precise control over structural parameters like porosity and strand alignment, making it ideal for personalized periodontal treatments and advanced tissue defect repair. Its compatibility with diagnostic imaging further enhances its utility in tailored therapies. However, high costs, energy demands, and the need for trained operators remain challenges to its widespread adoption in medical applications [195].

In esthetic procedures, such as gingivectomy or smile design, 3D-printed surgical guides have gained popularity. These guides, customized based on the patient’s digital scans, provide precise templates for gingival recontouring, ensuring predictable and esthetically pleasing outcomes. Fabricated using transparent biocompatible methacrylate-based resins in SLA or DLP printers, surgical guides offer high precision and clarity, making them ideal for anterior gingival surgeries [189,190].

## 4. Future Directions and Challenges in 3D-Printed Resin Materials for Dentistry

While 3D-printed resin materials offer significant innovations in dentistry, several challenges warrant attention for future advancements. The mechanical durability and biocompatibility of the current resins may not always meet the demands of the oral environment. Therefore, developing stronger, more durable, biocompatible resins is necessary for future advancements. The lack of standardized testing protocols and regulatory guidelines for 3D-printed dental materials may limit clinical applications. Establishing comprehensive standards is critical to ensure the safety and efficacy of these materials. The high costs of advanced 3D printing technologies can restrict their use, especially in resource-limited regions. Developing cost-effective materials and technologies will make these innovations more accessible to a broader audience.

Future research should focus on developing novel resin formulations, integrating multi-material printing capabilities, and utilizing artificial intelligence to optimize printing workflows. Additionally, comprehensive in vivo studies assessing the long-term clinical performance of 3D-printed dental materials are crucial for validating their efficacy and safety. Addressing these challenges and adopting innovative solutions will further advance the use of 3D-printed resin materials in dentistry, enhancing their clinical utility and improving patient outcomes.

## 5. Conclusions

The advancements in 3D printing technology, particularly in developing resin-based materials, have revolutionized modern dentistry by offering precise, customizable, and efficient solutions. However, 3D-printed resins must be biologically and mechanically evaluated through both in vitro and in vivo testing within a structured risk management process. While manufacturers provide usage guidelines, the responsibility for adhering to these instructions ultimately lies with the dentist. Nonetheless, the production process affects the biocompatibility and durability of the material, and compliance with the standards set for material properties is vital. Resin materials have become indispensable for various clinical applications with superior esthetic, mechanical, and biocompatible properties. These innovations have enabled more accurate treatment outcomes, reduced treatment times, and improved patient satisfaction. Ongoing research and technological developments are expected to further enhance resin materials’ properties, such as strength, translucency, and durability, thereby expanding their clinical applicability. The continued evolution of 3D printing in dentistry is anticipated to play a pivotal role in advancing patient-specific treatments, improving clinical workflows, and shaping the future of dental care.

## Figures and Tables

**Table 1:** Comparison of additive and subtractive manufacturing processes [7,8,17,19].

Property	Subtractive Manufacturing (Milling)	Additive Manufacturing (3D Printing)
**Material Waste**	Significant waste, such as excess material, is removed during the milling process and is often not reusable	Minimal waste due to layer-by-layer fabrication. Reported material savings of up to 95–98%, with unused resin and powder being recyclable
**Time Efficiency**	Time-intensive for intricate designs due to the removal of excess material and the need for post-processing	Faster for complex designs, mainly when producing multiple items simultaneously (e.g., up to 20 restorations printed in one session)
**Production Costs**	Higher costs due to material waste, expensive milling blocks, and longer production times	Lower equipment and consumable costs; up to 8–10 times cheaper for materials such as composite resin compared to milled PMMA or lithium disilicate
**Customization**	Limited customization; additional complexity increases production time and costs	High customization capability with minimal additional cost for unique designs and patient-specific applications
**Environmental Impact**	Higher environmental impact due to excessive material waste and higher energy requirements during milling	Reduced environmental footprint due to lower material consumption and energy use in production. Improved sustainability through local production

**Table 2:** Comparison of the advantages, disadvantages, costs, and production times of vat photopolymerization and material jetting [22,30,58].

	Techniques	
	Vat Photopolymerization	Material Jetting
**Advantages**	High resolution Good accuracy Smooth surfaces Suitable for producing complex geometries Cost-effective and relatively fast technology	High-resolution and surface quality Multi-material printing and combining multiple materials with different hardness and colors Esthetic suitability Colorful prototypes and models
**Disadvantages**	Material fragility Sensitivity to UV light and heat Post-processing requirements Limited material options	Durability issues (Photopolymers tend to degrade over time, losing their mechanical properties) Maintenance needs
**Cost**	Lower equipment and material costs (especially for SLA) Post-processing requirements can increase labor costs	Higher equipment and material costs
**Production Time**	Faster due to the light source curing entire layers at once (especially in DLP)	Longer due to more complex processes for creating detailed and esthetic models

**Table 3:** Commercially available 3D-printed photopolymer resins, production technologies, application areas, and manufacturers.

Material	Technology	Application	Manufacturer
Formlabs Permanent Crown Resin^®^	SLA	Permanent crown	Formlabs Inc., Somerville, MA, USA [80,81,82,83,84,85,86,87,88,89]
VarseoSmile Crown Plus	SLA, DLP	Permanent crown	Bego, Bremen, Germany [90,91,92,93,94,95]
CROWNTEC	DLP	Permanent crown	Saremco Dental AG, Rebstein, Switzerland [86,89,91,94,96,97]
NexDent C&B MFH	SLA	Temporary crown	Vertex-Dental B.V., Soesterberg, The Netherlands [82,90,98,99,100]
Detax Freeprint Temp	DLP	Temporary crown	Detax GmbH, Ettlingen, Germany [81,98,101,102,103]
GC TempPrint	DLP	Temporary crown	GC Corporation, Tokyo, Japan [81,103]
Formlabs Temporary CB	SLA	Temporary crown	Formlabs Inc., Somerville, MA, USA [81,84,103,104]
Graphy tera harz TC-80DP	DLP	Temporary crown	Graphy Inc., Seoul, Republic of Korea [82,98,102,105]
P Pro Crown& Bridge	DLP	Temporary crown	Straumann Cares, Basel, Switzerland [106]
Prov.Crown&Bridge	DLP	Temporary crown	Voco, Cuxhaven, Germany [106]
3Delta temp	DLP	Temporary resin	Deltamed, Friedberg, Germany [98]
Zenith ZMD-1000B^®^	DLP	Temporary resin	Dentis Co., Daegu, Republic of Korea [102]
Veltz DT-1 Temporary Teeth^®^	DLP	Temporary resin	Hephzibah, Incheon, Republic of Korea [102]
Resina 3D Smart Print Bio Denture	DLP	Denture base	SmartDent, São Paulo, Brasil [107]
Formlabs Denture Base RP	SLA	Denture base	Formlabs Inc., Somerville, MA, USA [108,109,110]
ASIGA DentaBASE	DLP	Denture base	ASIGA, Erfurt, Germany [108,111]
NextDent Denture 3D+	DLP	Denture base	NextDent B.V., Soesterberg, The Netherlands [111,112]
Cosmos Denture	DLP	Denture base	Yller Biomateriais AS, Pelotas, RS, Brazil [112]
Formlabs Denture Teeth	SLA	Denture teeth	Formlabs Inc., Somerville, MA, USA [104]
OnX	DLP	Denture teeth	Sprintray, Los Angeles, CA, USA [95]
Flexcera Ultra+	DLP	Denture teeth	Desktop Health, Burlington, MA, USA [95]
Dima Print Denture Teeth^®^	DLP	Denture teeth	Kulzer GmbH, Hanau, Germany [102]
Detax IBT resin	DLP	Indirect bonding tray	Detax GmbH, Ettlingen, Germany [113]
Formlabs IBT resin	SLA	Indirect bonding tray	Formlabs, Ohio, Milbury, OH, USA [114,115]
TEC resin	DLP	Digital align dental model	Detax GmbH, Ettlingen, Germany [113]
Hard Model 2.0 resin	DLP	Digital align dental model	Nextdent B.V., Soesterbeg, The Netherlands [116]
Tera Harz TC-85 DAC (Direct aligner clear)	DLP	Aligner	Graphy, Seoul, Republic of Korea [117,118,119]
Tera Harz TC85A aligner resin	DLP	Aligner	Graphy, Seoul, Republic of Korea [120,121,122,123,124,125]
Tera Harz TC-85 DAW (Direct aligner white)	DLP, LCD	Aligner	Graphy, Seoul, Republic of Korea [126]
Material X	DLP	Aligner	Envisiontec, Inc.; Dearborn, Mich [127]
OD-Clear TF	DLP	Aligner	3DResyns, Barcelona, Spain [127]
SprintRay Die resin	DLP	Model resin	SprintRay, Los Angeles, CA, USA [124]
Gray model resin	DLP	Model resin	SprintRay, Los Angeles, CA, USA [124]
Dental Model V2, Formlabs	SLA	Dental model	Formlabs Inc., Somerville, MA, USA [128,129,130]
NextDent Model 2.0	DLP	Dental model	NextDent B.V., Soesterberg, The Netherland [129,131]
3D acrylic resin	DLP	Molding plate	Vertex-Dental B.V., Soesterberg, The Netherlands [132]
NextDent OrthoFlex	DLP	Dental splints, retainers	NextDent B.V., Soesterberg, The Netherland [133]
Dental LT Clear Resin	SLA	Splints, occlusal guards, orthodontic appliances, retainer	Formlabs Inc., Somerville, MA, USA [115,133,134]
VisiJet Crystal	Multijet	Replica of the tooth (for education)	3D Systems, Rock Hill, SC, USA [135,136]
VeroWhitePlusTM	PolyJet	Replica of the tooth (for education) Surgical guide	Stratasys Ltd., Eden Prairie, MN, USA [65,137]
Visijet M3 Stoneplast	Multijet	Composite Injection Guides	3D Systems, Rock Hill, SC, USA [61,138,139]
Anycubic Clear UV Resin	DLP	3D-printed gingivectomy and Tooth reduction guide	Anycubic, Shenzhen, China [64,140]
NextDent SG	DLP	Endodontic guide Surgical guide	Nextdent B.V., Soesterberg, The Netherlands [62,141,142]
MED610	PolyJet	Surgical guide	Stratasys Ltd., Minneapolis, MN, USA [143]
DentalSG	SLA	Surgical guide	Formlabs Inc., Somerville, MA, USA [137,141,142,144,145]
NextDent Ortho Clear	DLP	Surgical guide	Nextdent B.V., Soesterberg, The Netherlands [134]
BioMed Amber resin	SLA	Surgical guide	Formlabs, Ohio, Milbury, OH, USA [114,115]
SHERAprint-SG	DLP	Surgical guide	SHERA Werkstoff-Technologie GmbH & Co. KG, Lemforde, Germany [137,142]
V-PrintSG	DLP	Surgical guide	Voco, Cuxhaven, Germany [142]
Clear Resin	SLA	Surgical guide	Formlabs Inc., Somerville, MA, USA [146]
Dima Print Guide	DLP	Surgical guide	Kulzer GmbH, Hanau, Germany [137]
Asiga DentaCLEAR	DLP	Surgical guide	Asiga, Alexandria, NSW, Australia [147]
Asiga DentaGUIDE	DLP	Surgical guide	Asiga, Alexandria, NSW, Australia [148]
Rigid 10K Resin	SLA	Resin die	Formlabs Inc., Somerville, MA, USA [93]

## Data Availability

Not applicable.

## Abbreviations

The following abbreviations are used in this manuscript:

ASTM	American Society for Testing and Materials
CAD–CAM	Computer-Aided Design–Computer-Aided Manufacturing
CLIP	Continuous Liquid Interface Production
DLP	Digital Light Processing
mSLA	Masked Stereolithography
PEEK	Polyetherretherketone
PMMA	Polymethylmethacrylate
SLA	Stereolithography
STL	Standard Tessellation Language
UV	Ultraviolet
